# 0727. Normal saline versus ringer´s lactate in experimental sepsis

**DOI:** 10.1186/2197-425X-2-S1-P49

**Published:** 2014-09-26

**Authors:** D Orbegozo Cortes, S Fuhong, C Santacruz, K Hosokawa, H Xinrong, J Creteur, J-L Vincent, D De Backer

**Affiliations:** Dept of Intensive Care, Erasme Hospital, Brussels, Belgium

## Introduction

The development of hyperchloremic acidosis associated with normal saline (NS) administration may be deleterious in septic shock.

## Objectives

To compare the time-course of hemodynamics, organ dysfunction and survival with NS or Ringer's lactate (RL) in an experimental model of severe peritonitis.

## Methods

Fourteen adult sheep (24-34 Kg) were anesthetized (midazolam, ketamine and morphine), mechanically ventilated and invasively monitored. A cecotomy was performed to collect autologous feces that were later re-injected into the peritoneal cavity through the abdominal wall to create abdominal sepsis. RL was administered during the surgical procedure. After baseline measurements, animals were randomly allocated to receive only NS or RL titrated to maintain pulmonary artery occlusion pressure at baseline level. Neither vasoactive agents nor antibiotics were used during the experiment. Animals were followed until death or for a maximum of 30 hours. Time-evolution for repeated measurement data was analyzed using a Generalized Estimating Equations approach in SPSS 19.0 (IBM) with a p < 0.05 considered as significant. Data are presented as median with inter-quartile range.

## Results

See tables 1, 2 and 3

**Table 1:** Acid-base parameters

		T0	T4	T8	T12	T16	T20	T24	T28
**Arterial pH**	**RL**	7.41 (7.38-7.44)	7.43 (7.39-7.45)	7.41 (7.36-7.43)	7.37 (7.36-7.38)	7.33 (7.32-7.38)	7.24 (7.18-7.27)	7.12 (7.06-7.18)	7.12 (6.87-7.38)
	**NS**	7.40 (7.36-7.49)	7.39 (7.36-7.40)*	7.35 (7.25-7.35)*	7.23 (7.14-7.29)*	7.03 (6.97-7.18)*	7.10 (7.02-7.18)*	6.99 (6.99-6.99)*	-
**Arterial PCO2 (mmHg)**	**RL**	36 (32-36)	31 (30-31)	32 (30-32)	33 (31-35)	33 (32-34)	35 (34-37)	35 (32-36)	41 (32-50)
	**NS**	34 (33-37)	32 (30-34)	32 (31-34)	32 (31-34)	35 (33-36)	34 (33-34)	33 (33-33)	-
**Chloride (mmol/L)**	**RL**	108 (105-109)	110 (106-111)	111 (108-112)	111 (109-114)	113 (112-114)	114 (113-117)	112 (112-113)	110 (109-110)
	**NS**	111 (107-113)	115 (112-117)*	121 (117-123)*	126 (122-128)*	127 (124-130)*	128 (125-130)*	126 (126-126)*	-
**Arterial Lactate (mmol/L)**	**RL**	1.2 (1.0-1.5)	1.2 (0.9-1.5)	1.4 (1.1-1.7)	1.4 (1.2-1.8)	2.0 (1.3-3.3)	3.7 (3.2-5.4)	6.6 (5.9-10.0)	6.0 (1.1-10.8)
	**NS**	1.3 (0.8-1.9)	0.8 (0.7-1.2)	1.1 (0.7-1.3)*	1.5 (1.1-2.1)	5.8 (1.2-7.3)*	3.6 (2.3-4.8)	5.7 (5.7-5.7)	-

* = p < 0.05 compared with RL group

**Table 2:** Systemic parameters

		T0	T4	T8	T12	T16	T20	T24	T28
**Mean Arterial Pressure (mmHg)**	**RL**	103 (97-111)	90 (77-94)	82 (77-90)	68 (60-85)	59 (55-79)	55 (54-57)	51 (39-57)	36 (26-46)
	**NS**	97 (93-109)	88 (79-93)	67 (63-81)*	52 (41-63)*	37 (33-42)*	46 (36-57)*	40 (40-40)*	-
**Cardiac Index (L/min/m²)**	**RL**	5.1 (4.0-5.6)	4.4 (3.9-6.6)	4.7 (3.9-5.1)	4.6 (3.4-6.0)	4.3 (3.5-5.4)	4.9 (3.5-5.9)	4.6 (2.7-5.0)	3.9 (3.0-4.7)
	**NS**	4.9 (4.0-5.5)	4.8 (3.8-5.5)	3.2 (2.6-4.2)*	2.9 (2.7-3.3)*	2.1 (1.5-2.8)*	3.1 (2.9-3.3)*	1.4 (1.4-1.4)*	-
**Diuresis (ml/Kg/H)**	**RL**	1.0 (0.5-2.0)	0.9 (0.6-2.2)	1.3 (0.5-2.3)	1.2 (0.3-2.9)	0.2 (0.0-1.3)	0.1 (0.0-0.4)	0.0 (0.0-0.0)	0.5 (0.0-1.0)
	**NS**	1.0 (0.6-1.8)	0.9 (0.7-1.2)	1.3 (0.2-2.8)	0.2 (0.0-0.4)*	0.0 (0.0-0.1)	0.2 (0.0-0.4)	0.0 (0.0-0.0)	-
**PaO2/FiO2 ratio**	**RL**	462 (420-493)	482 (364-496)	458 (317-483)	352 (222-444)	266 (220-359)	251 (170-324)	281 (150-298)	138 (64-212)
	**NS**	388 (322-420)	385 (284-486)	268 (259-400)*	192 (173-299)	176 (160-292)	217 (177-257)	204 (204-204)	-

* = p < 0.05 compared with RL group

**Table 3:** Laboratory parameters

		T0	T4	T8	T12	T16	T20	T24	T28
**Creatinine (mg/dL)**	**RL**	0.9 (0.7-1.2)	0.8 (0.6-1.0)	0.9 (0.6-1.0)	1.1 (0.7-1.2)	1.4 (0.9-2.0)	2.0 (1.4-2.7)	3.0 (2.4-3.2)	2.3 (1.4-3.2)
	**NS**	0.7 (0.6-1.0)	0.7 (0.6-0.8)	0.7 (0.7-0.9)	1.2 (0.8-1.6)	2.0 (1.1-2.5)	1.5 (1.4-1.6)	2.4 (2.4-2.4)	-
**PTT (sec)**	**RL**	32 (28-36)	33 (32-41)	40 (35-47)	45 (40-53)	49 (40-59)	50 (40-61)	60 (49-70)	108 (67-150)
	**NS**	36 (24-42)	34 (27-42)	45 (37-47)	55 (46-55)	62 (50-135)	104 (58-150)*	63 (63-63)	-

* = p < 0.05 compared with RL group

Survival time was significantly shorter in the NS group than in the RL group (17 [14-20] hours vs 26 [23-29] hours, p Logrank=0.003).

## Conclusions

In this sheep model of severe abdominal sepsis, NS-induced hyperchloremic acidosis was associated with an increased development of organ dysfunction and greater mortality.

